# Measuring adherence to antiretroviral treatment in resource-poor settings: The clinical validity of key indicators

**DOI:** 10.1186/1472-6963-10-42

**Published:** 2010-02-19

**Authors:** Dennis Ross-Degnan, Marsha Pierre-Jacques, Fang Zhang, Hailu Tadeg, Lillian Gitau, Joseph Ntaganira, Robert Balikuddembe, John Chalker, Anita K Wagner, INRUD IAA

**Affiliations:** 1 Department of Population Medicine, Harvard Medical School and Harvard Pilgrim Health Care Institute, Boston MA, USA; 2 Management Sciences for Health, Addis Ababa, Ethiopia; 3 Sustainable Health Care Foundation, Nairobi, Kenya; 4 Department of Epidemiology, School of Public Health, National University of Rwanda, Kigali, Rwanda; 5 Department of Clinical Pharmacology, Makerere University, Kampala, Uganda; 6 Center for Pharmaceutical Management, Management Sciences for Health, Arlington VA, USA; 7 International Network for the Rational Use of Drugs Initiative on Adherence to Antiretrovirals, Management Sciences for Health, Arlington, VA, USA

## Abstract

**Background:**

Access to antiretroviral therapy has dramatically expanded in Africa in recent years, but there are no validated approaches to measure treatment adherence in these settings.

**Methods:**

In 16 health facilities, we observed a retrospective cohort of patients initiating antiretroviral therapy. We constructed eight indicators of adherence and visit attendance during the first 18 months of treatment from data in clinic and pharmacy records and attendance logs. We measured the correlation among these measures and assessed how well each predicted changes in weight and CD4 count.

**Results:**

We followed 488 patients; 63.5% had 100% coverage of medicines during follow-up; 2.7% experienced a 30-day gap in treatment; 72.6% self-reported perfect adherence in all clinic visits; and 19.9% missed multiple clinic visits. After six months of treatment, mean weight gain was 3.9 kg and mean increase in CD4 count was 138.1 cells/mm3.

Dispensing-based adherence, self-reported adherence, and consistent visit attendance were highly correlated. The first two types of adherence measure predicted gains in weight and CD4 count; consistent visit attendance was associated only with weight gain.

**Conclusions:**

This study demonstrates that routine data in African health facilities can be used to monitor antiretroviral adherence at the patient and system level.

## Background

Global health initiatives have introduced antiretroviral therapy (ART) to ever-increasing numbers of HIV patients. Successful therapy depends on life-long adherence to these medications. Thus far, large-scale African ART program have reported mixed results on patient adherence to antiretrovirals (ARVs),[1] with some programs reporting high levels,[2,3] and some reporting much lower levels[4]. With rapidly expanding access to ARVs in resource-poor settings, it will be vital to monitor adherence and to identify interventions that can encourage sustained adherence.

Accurate assessment of adherence is critical to maximize clinical efficacy and minimize the potential population risks associated with drug resistance. However, no validated approaches exist to measure adherence, especially in low resource settings with potentially poor data availability.

Patient self-report about recent adherence is a common assessment method due to its relative ease and low cost of data collection, but self-reports tend to overestimate adherence[5,6]. In addition, self-report adherence measures have been operationalized in different ways[7]. A recent meta-analysis showed that self-report adherence measures are predictive of clinical outcomes,[8] a finding that has been replicated in resource poor settings [2-4,9]. However, no studies have validated whether routine self-report data in medical or pharmacy records are predictive of clinical outcomes.

Pill counts, like self-reports, can overestimate adherence when compared with electronic medication monitoring[5,6]. Collecting pill count data requires a separate recording process in the pharmacy that is often not part of routine dispensing operations. Nevertheless, pill counts have also been shown to be associated with viral load and CD4 counts[6].

Pharmacy refill records are commonly used in settings with electronic pharmacy data systems to calculate adherence indicators,[10,11] either percentage of days within a defined period covered by medicines dispensed or occurrence of gaps between dispensings. Several studies have shown associations between dispensing-based adherence measures and clinical outcomes, including viral load and CD4 counts[12,13]. Pharmacy refill approaches have not been extensively tested in settings with manual dispensing records, where data completeness and quality may be problematic.

Consistency of clinic attendance is potentially another way to assess continuity of care and risk for poor adherence. Because failure to attend clinic when expected is objective and easy to ascertain in most record systems, inconsistency of attendance may identify patients in need of outreach or adherence counseling.

In 2006 the International Network for Rational Use of Drugs (INRUD) and national HIV/AIDS programs in five East African countries began the five-year Initiative on Antiretroviral Adherence (IAA) to develop practical interventions to improve adherence to ART in routine treatment settings. They found wide variations in definitions and practice in measuring and reporting adherence[14]. To address this gap, the INRUD-IAA group has developed and pilot tested methods and indicators to assess adherence at health facilities using patient interviews and the types of routine data available in these settings, which are reported in a companion publication[15]. These indicators can be used to measure the success of health facilities in maintaining patients on treatment and to evaluate the impact of interventions.

This study was designed to assess the correlation between several of the INRUD-IAA measures: patient self-reports and pill counts (documented in clinical and pharmacy records); pharmacy dispensing-based indicators; and attendance consistency. To validate these measures, we also assessed the extent to which they predict changes in weight and CD4 count 5-14 months after start of ART in treatment naïve patients.

## Methods

### Overall study design

We conducted a retrospective cohort study in 16 HIV/AIDS treatment facilities in Ethiopia, Kenya, Rwanda, and Uganda using data from medical and pharmacy records on attendance, medication dispensing, pill counts or self-report adherence, demographic and clinical covariates, and clinical outcomes.

### Facility sample

From 80 facilities included in our pilot studies to test adherence indicators, [as referenced above] we selected four per country to represent the range of infrastructures and patient populations. Facilities that primarily conduct AIDS clinical research were excluded and no more than one referral center was included in each country.

### Patient sample

The study sample targeted 30 treatment naïve patients per facility who initiated ART 8-24 months prior to data collection and who met the following inclusion criteria: (a) ≥ 18 years old; (b) no previous exposure to ART except for prevention of mother to child transmission (PMTCT); (c) follow-up data for ≥ 6 months after ART initiation, as indicated by one or more recorded clinic visits in two consecutive 3-month periods after initiation; (d) at least one adherence self-report or pill count in medical or pharmacy records in these two quarters; (e) data available on prescribed ART regimen and quantity dispensed during the follow-up period; (f) at least one CD4 count recorded within 3 months prior to initiation and in the 18 months after initiation.

### Data collection and variables

Trained data collectors abstracted data from patient intake records, appointment schedules, and medical and pharmacy records for 18 months following ART initiation or until the date of data collection, whichever occurred first.

All data for measuring patient adherence were extracted from routine clinic records, where they had been recorded by the treating clinician, nurse, or pharmacist during the clinic visit. Data for calculating adherence measures included: self-reported adherence; pill counts, documented as number of days of therapy remaining from previous dispensings; and type and days of antiretroviral medicines dispensed. Self-reported adherence was typically documented in medical records as perfect (which we coded as 1), good (2), or poor (3). In Ethiopia, self-reports were documented as good/fair vs. poor; because of the difference in coding, cases from Ethiopia were excluded from statistical analyses of self-reports. We calculated consistency of clinic attendance using scheduled and actual visit dates. Pill counts were recorded for <38% of clinic visits and the information recorded varied, so we dropped pill count adherence from the analysis.

We constructed the following adherence measures at every follow-up visit after ART initiation: (a) average self-reported adherence from initiation to visit date; (b) whether the patient ever self-reported poor adherence; (c) percentage of days since ART initiation covered by dispensed medicines; (d) whether the patient ever experienced a gap of >30 days without ARVs, assuming daily consumption until available supply was exhausted; (e) percentage of visits to date that occurred on or before the scheduled date and the percentage within 3 days after the scheduled date; and (f) percentage of visits that occurred on or before the expected date of finishing the ARVs dispensed in the previous visit (usually 30 days supply). The last indicator was developed as an alternative to the attendance-based indicators to accommodate systems where data on the dates of scheduled visits are not readily available.

Health facility information collected included type (national, provincial, district, or other hospital, or health center), location (capital, other urban, or rural area), and management (public, private, mission, other non-governmental organization). Patient demographic information included age, gender, marital status, living situation, education, occupation, and presence of a treatment support partner. Clinical information included WHO stage of HIV/AIDS on ART initiation, evidence of ARVs to prevent mother to child transmission, evidence of tuberculosis at ART initiation, duration of ART, and ART regimen prescribed at each visit. We also extracted symptoms or diagnoses that might indicate possible side effects, opportunistic infections, or significant clinical events associated with HIV.

Our primary outcomes were weight and CD4 counts. We recorded weight in kilograms at baseline and at every subsequent visit, and calculated weight gain (or loss) since the initiation of therapy at each visit. Treatment protocols typically call for patients to have a follow-up CD4 test within the first 6 months after initiating ART, although because of limitations in laboratory availability, this may stretch to a longer interval. We captured the dates and results of all CD4 tests from 3 months prior to initiating ART until the end of follow-up. For patients with at least one CD4 count recorded between 4 and 9 months after initiation of ART, we calculated changes between the baseline and the date of the follow-up CD4 count.

### Data management and analysis

Country data collectors entered data twice into standardized Excel spreadsheets; data entry differences and data errors were resolved by study staff. Using Pearson product moment coefficients (for continuous measures) and Kendall tau rank correlation coefficients (when one or both measures were binary), we estimated correlations among the different adherence and visit measures.

We used generalized estimating equations (GEE) to predict weight change from baseline to each clinic visit during the first 270 days after ART initiation and ordinary least squares regression models to predict change in CD4 count from baseline to the follow-up CD4 test. Each adherence indicator was modeled separately for each clinical outcome; because of the difference in the way the indicator was reported, patients from Ethiopia were excluded from analyses modeling average self-reported adherence. All models included: gender, age (30 or younger, 31-40, >40 years), whether married, capital-urban-rural clinic location, baseline CD4 level, WHO stage at ART initiation (stage 1 or 2 vs. stage 3 or 4), time since ART initiation, evidence of TB at ART initiation, evidence of side effects or opportunistic infections since treatment initiation, number of different ART regimens, and whether the patient was ever treated with a protease inhibitor; weight change models also included baseline weight. All analyses were performed in SAS version 9.1 (SAS Institute, Cary, NC).

### Ethical approval

This study was approved by the AIDS Control Programs of the participating countries and by the Harvard Pilgrim Health Care Human Studies Committee.

## Results

### Demographic and clinical characteristics

The overall sample consisted of 488 patients. Of patients screened for sample inclusion, 7.4% were under age 18, 3.9% had evidence of previous ART; 4.8% did not have clinic visits in each of the first two quarters after initiating ART, and 8.6% did not have adherence self-reports in the first two post-initiation quarters. Gender, age, and WHO stage at ART initiation were similar between included and excluded patients.

Table 1 describes key demographic and clinical characteristics of the overall study group and the subgroup having a CD4 test within the target window of 91 to 270 days after treatment initiation. About 63% of patients were women and average age was 36 years (SD = 8.3). Half had a primary school education and another third had attended secondary school or college; about 40% were currently unemployed. The sample was predominantly treated in urban health facilities (76%), nearly two-thirds in government facilities. About half of patients were WHO stage 3 when initiating ART and another 25% initiated treatment at stage 2. In the baseline CD4 test, 37% of patients had CD4 counts of ≤ 100, 40% had counts of 101-200, and 23% were ≥ 201. Males had an average baseline weight of 57.2 kg (SD = 9.7), while women averaged 52.9 kg (SD = 9.2).

**Table 1:** Demographic and clinical characteristics of patients in the overall study cohort and in the subgroup with follow-up CD4 tests 4 to 9 months after ART initiation.

	**Total** **(n = 488)**	**With CD4 test** **(n = 409)**
		
Female	62.5%	63.1%
Age		
30 & under	29.3%	29.6%
31-40	43.4	42.8
41 & over	27.3	27.6
Education		
None	15.5%	16.8%
Primary	46.7	47.3
Secondary or greater	37.8	36.0
Married currently	44.1%	44.9%
Living		
Alone	6.0%	6.1%
With child only	24.5	23.8
With adult +/- child	69.6	70.1
Occupation		
Employed	28.3%	28.5%
Self-employed	32.2	32.0
Unemployed	39.4	39.5
Treated in government facility	63.5%	61.4%
Treatment location		
Clinic in capital	49.8%	50.4%
In other urban area	26.4	25.4
In rural area	23.8	24.2
WHO stage at initiation		
Stage 1	8.9%	7.9%
Stage 2	25.4	26.5
Stage 3	54.4	53.3
Stage 4	11.3	12.3
Has treatment support partner	88.7%	89.7%
Evidence of previous PMTCT (female only)	6.2%	4.7%
Evidence of current or prior TB at initiation	15.2%	13.2%
Baseline CD4 value		
< = 100	37.4%	36.7%
101-200	40.4	42.5
201-350	20.9	19.3
351 an above	1.6	1.4
Weight in kg (SD) at initiation		
Males	57.2 (9.2)	57.1 (9.2)
Females	52.9 (9.7)	52.9 (9.8)

Table 2 presents the values of the main study adherence, attendance, and clinical outcome measures at the time of follow-up measurement. Patients generally maintained high rates of adherence to therapy in the first six to nine months of treatment. Over 83% had ART coverage rates greater than 95% and another 14% had coverage rates from 80% to 95%. About 3% of patients had coverage rates less than 80% or experienced a gap in therapy of ≥ 30 days during the follow-up period.

**Table 2:** Adherence and clinical outcome measures at visit nearest to 180 days after initiation or at second CD4 test in subgroup with test in months 4 to 9.

	**Total** **(n = 488)**	**With CD4 test** **(n = 409)**
		
Average number of days since treatment initiation (min, max)	178 (124,337)	188 (98,270)
**Dispensing-based adherence**		
% of days covered since ART start		
< 80%	2.9%	2.4%
80% to <85%	2.7	2.0
85% to <90%	2.9	3.9
90% to <95%	8.0	8.1
95% to <100%	20.1	19.6
100%	63.5	64.1
% with gap in treatment >30 days	2.7%	3.4%
**Self-report adherence**		
Average (s.d.) number of self-reports recorded in medical record during follow-up	5.7 (1.8)	5.4 (2.0)
Average (s.d.) of all self-reports *	1.17 (0.39)	1.16 (0.33)
% all self-reports perfect *	72.6%	74.4%
% any self-report poor *	3.0%	2.9%
**Appointment keeping**		
Average number (s.d.) of visits	6.4 (1.1)	6.0 (1.6)
% visits on/before scheduled day		
< 80%	19.9%	18.1%
80% to <90%	22.1	20.5
90% to 100%	58.1	61.4
% visits ≤ 3 days after scheduled		
< 80%	5.9%	5.7%
80% to <90%	13.1	11.1
90% to 100%	81.0	83.2
**Dispensing-based appointment keeping**		
% of visits before finishing ARVs dispensed during previous visit		
< 80%	44.9%	44.0%
80% to <90%	27.5%	27.1
90% to 100%	27.7%	28.9
**Change in clinical outcome measures**		
Average (s.d.) increase in CD4 count	NA	138.1 (125.5)
Average (s.d.) weight gain (kg)	3.9 (5.1)	3.8 (4.8)

* Self-reports were recorded in medical records as perfect (coded as 1), good (2), or poor (3); cases from Ethiopia (n = 120 total, n = 96 with CD4 test) were excluded from analysis because self-reports there were recorded as good/fair vs. poor.

Patient self-reports mirror the positive results of the dispensing adherence measures. The average self-reported adherence score was 1.17 (out of 3). Nearly three-quarters of patients (72.6%) reported perfect adherence in all clinic visits; only 3% of patients reported poor adherence in any visit.

Patients averaged more than one visit per month during follow-up and most attended a high percentage of visits as scheduled. About 20% of patients did not come on the day expected and only 6% failed to appear within 3 days for more than one in five of their visits. For the measure based on attending before all medicines dispensed in the previous visit were finished, consistency appears worse; 45% of patients did not come on or before the day on which medicines were finished for more than one in five of their visits. Since this measure does not account for medicines remaining from earlier visits, it may overestimate the frequency of running out of medicines.

Finally, ART initiation is clearly associated with improvements in clinical outcomes. Patients gained an average of 3.9 kg (SD = 5.1) in the first six months of treatment. In the subgroup with a follow-up CD4 test, the average improvement in CD4 count was 138 (SD = 125) cells/mm^3 ^during that period.

### Correlations among adherence measures

Table 3 reports correlations among the adherence measures. The patterns and overall strength of the correlations between the different measures suggest that they address related behavioral domains.

**Table 3:** Correlations among dispensing, self-report, and attendance-based adherence measures at time of clinic visit closest to 180 days after treatment initiation.

**Correlation Significance** **N** **(bold = p < 0.05)**	**Coverage >80%**	**Coverage >95%**	**Coverage gap >30 days**	Average self-report adherence *	Any self-report adhe-rence less than perfect*	% of visits on or before scheduled day**	% of visits within 3 days of scheduled day**	**% of visits before last dispensing finished**
								
% of days covered	**0.335** **< .0001** **488**	**0.744** **< .0001** **488**	**-0.321** **< .0001** **488**	**-0.186** **.0004** **358**	**-0.185** **.0004** **368**	**0.324** **< .0001** **458**	**0.357** **< .0001** **458**	**0.560** **< .0001** **488**
Coverage >80% &		**0.388** **< .0001** **488**	**-0.734** **< .0001** **488**	-0.002 .9762 358	0.010 .8474 368	**0.186** **< .0001** **458**	**0.305** **< .0001** **458**	**0.237** **< .0001** **488**
Coverage >95% &			**-0.374** **< .0001** **488**	**-0.178** **.0007** **358**	**-0.162** **.0018** **368**	**0.264** **< .0001** **458**	**0.376** **< .0001** **458**	**0.399** **< .0001** **488**
Coverage gap >30 days &				-0.003 .9480 358	-0.010 0.8471 368	**-0.120** **.0104** **458**	**-0.222** **< .0001** **458**	**-0.206** **< .0001** **488**
Average self-report adherence					**0.983** **< .0001** **358**	**-0.136** **.0104** **357**	**-0.160** **.0024** **357**	0.072 0.1725 358
Any self-report adherence less than perfect &						**-0.141** **.0069** **367**	**-0.164** **.0016** **367**	-0.044 0.3961 368
% visits on/before scheduled day							**0.630** **< .0001** **458**	**0.360** **< .0001** **458**
% visits within 3 days of scheduled day								**0.367** **< .0001** **458**

* Self-reports (n = 368) coded 1 = perfect, 2 = good, 3 = poor; excludes n = 120 cases from Ethiopia where self-reports were scored good/fair vs. poor

** n = 458 for appointment keeping variables due to missing scheduled visit dates

& Kendall tau rather than Spearman rank-order correlations are reported when these variables are correlated with each other

The dispensing-based coverage measures were all highly inter-correlated, which is not surprising since they are derived from the same data source. The dispensing-based measures are also highly correlated with the attendance-based measures, suggesting that patients who present regularly for appointments maintain a more continuous supply of medicines. Self-report measures (average self-report in prior visits or ever reporting less than perfect adherence) are both significantly correlated with percent of days covered and coverage >95%, as well as with regularly attending clinic on the scheduled day or within 3 days.

### Adherence predicting weight change

**Figure 1:**
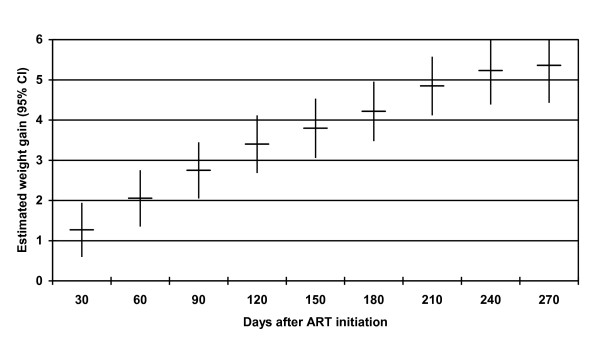
**Adjusted monthly weight gain in the first 9 months following initiation of ART**. Adjusted monthly weight gain in the first 9 months following initiation of ART in all patients in the sample estimated from generalized estimating equations. Model adjusts for gender, age category (30 or younger, 31-40, >40 years), whether married, capital-urban-rural location, baseline CD4 level, baseline weight, WHO stage at ART initiation (stage 1 or 2 vs. stage 3 or 4), evidence of TB at ART initiation, any evidence in medical record of side effect or opportunistic infection since treatment initiation, number of different ART regimens since initiation, and whether the patient was ever treated with a protease inhibitor.

**Figure 2:**
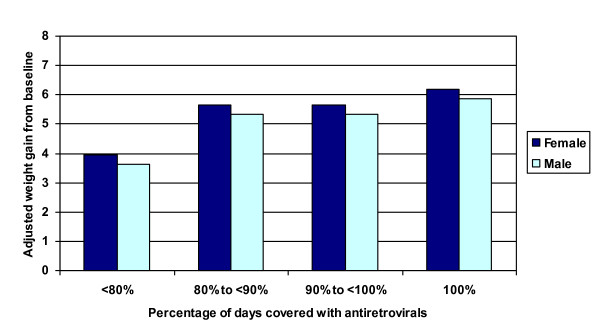
**Adjusted weight gain and adherence at nine months after initiation of ART**. Adjusted weight gain for a typical patient at the end of the nine-month period following initiation of ART by percentage of days covered with antiretroviral therapy. GEE model adjusts for gender, age category (30 or younger, 31-40, >40 years), whether married, capital-urban-rural location, baseline CD4 level, baseline weight, WHO stage at ART initiation (stage 1 or 2 vs. stage 3 or 4), evidence of TB at ART initiation, any evidence in medical record of side effect or opportunistic infection since treatment initiation, number of different ART regimens since initiation, ever treated with a protease inhibitor, and time since ART initiation. Typical patients were age 31-40, living in the capital, married, with baseline CD4 = 139.8, baseline weight = 52.8 kg for women and 57.2 kg for men, an average of 1.6 different ART regimens, no side effects or opportunistic infections during treatment, and never on PI.

Table 4 presents estimates from multivariate GEE models examining the relationship between the various adherence measures and weight gain. Controlling for demographic and clinical factors, the average adjusted monthly weight gain during the first 9 months of treatment was dramatic (Figure 1). The categorical dispensing-based coverage measure was significantly associated with greater weight gain during follow-up (p = 0.04 for inclusion of this variable in the model). Higher rates of medication coverage were associated with greater weight gain (Figure 2). Patients with <80% coverage gained significantly less weight than those who were more adherent, but weight gain did not differ significantly by degree of adherence over 80%. Based on adjusted estimates from the model, typical male patients in this cohort gained 5.9 kg by month 9 if perfectly adherent and 3.6 kg if <80% adherent, while female patients gained 6.2 kg and 3.9 kg respectively.

**Table 4:** Estimates from GEE models of indicators of adherence and appointment keeping as predictors of weight gain (in kilograms) in the first 9 months after ART initiation.

	Parameter Estimate	P-value	Confidence Interval
Coverage (vs. 100% coverage) *			
<80% of days covered	**-2.24**	**0.0012**	**-3.59, -0.89**
80% to <90% of days covered	-0.51	0.3517	-1.59, 0.57
90% to <100% of days covered	-0.52	0.1504	-1.23, 0.19
Gap >30 days (vs. no gap in coverage)	**-3.26**	**0.0010**	**-5.20, -1.31**
Average self-report (vs. all self-reports perfect) ** &			
Better than good but less than perfect	**-1.06**	**0.0368**	**-2.05, -0.06**
Good or worse	**-1.23**	**0.0352**	**-2.38, -0.09**
Any self-report less than perfect (vs. all perfect) &	**-1.14**	**0.0062**	**-1.96, -0.32**
<80% of visits on day scheduled	**-0.72**	**0.0413**	**-1.41, -0.03**
<80% of visits within 3 days of schedule	**-1.38**	**0.0127**	**-2.47, -0.29**
<80% of visits before medicines finished	**-0.71**	**0.0140**	**-1.28, -0.14**

Key: All models use variables measured on the day of each follow-up visit and include all visits up to 270 days following ART initiation. For all models except those involving self-reported adherence, n = 479 cases and n = 3384 follow-up clinic visits; for self-report adherence models, n = 354 cases and n = 2404 follow-up clinic visits. Models include: gender, age category (30 or younger, 31-40, >40 years), whether married, capital-urban-rural location, baseline CD4 level, baseline weight, WHO stage at ART initiation (stage 1 or 2 vs. stage 3 or 4), time since ART initiation (in nine 30-day categories up to 270 days), evidence of TB at ART initiation, any evidence in medical record of side effect or opportunistic infection since treatment initiation, number of different ART regimens since initiation, ever treated with a protease inhibitor.

* GEE type 3 score statistic for inclusion of 3 coverage terms in model, p = 0.0358

** GEE type 3 score statistic for inclusion of 2 self-report terms in model, p = 0.0331

& Models exclude cases from Ethiopia which assessed self-reported adherence differently

A total of 13 patients experienced a gap in therapy of >30 days during follow-up. Average medication coverage for these patients was 53% compared to 98% for the remaining cohort members. Based on the controlled GEE models, patients experiencing a 30-day gap in treatment gained an estimated 3.3 kg less (-5.2, -1.3) than those who did not experience such a gap, averaged across all visits during the follow-up period.

A self-report of less than perfect adherence during any clinic visit since initiation of therapy was significantly associated with gaining 1.1 kg less (-2.0, -0.3) than those who always self-reported perfect adherence. Average self-reported adherence during all prior visits was also significantly related to weight gain. Compared to patients reporting perfect adherence in all visits, patients who averaged between good and perfect self-reported adherence gained 1.1 kg less (-2.1, -0.1), and patients who averaged good adherence or worse gained 1.2 kg less (-2.4,-0.1).

All measures of consistent clinical attendance were significantly associated with weight gain. Based on GEE models, patients who attended <80% of their visits within 3 days of the scheduled day experienced a 1.4 kg lower (0.3, 2.5) weight gain than those who attended a greater percentage of their visits on time.

### Adherence predicting change in CD4 count

**Figure 3:**
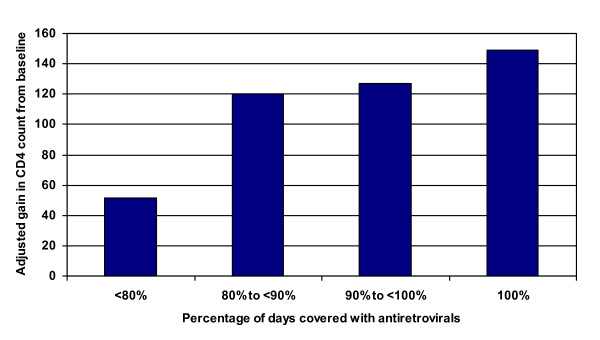
**Adjusted gain in CD4 count and adherence at 4 to 9 months after initiating ART**. Adjusted gain in CD4 count at a follow-up CD4 test conducted 4 to 9 months after initiating therapy by percentage of days covered with antiretroviral therapy. Linear regression model adjusts for gender, age category (30 or younger, 31-40, >40 years), whether married, capital-urban-rural location, baseline CD4 level, WHO stage at ART initiation (stage 1 or 2 vs. stage 3 or 4), evidence of TB at ART initiation, any evidence in medical record of side effect or opportunistic infection since treatment initiation, number of different ART regimens since initiation, ever treated with a protease inhibitor, and time since ART initiation. Estimates were adjusted based on the distribution of these characteristics in the overall population included in the model.

Table 5 presents results from the linear regression models examining the adherence measures as predictors of changes in CD4 count. As with weight gain, the dispensing-based adherence measures were significantly associated with changes in CD4 count during follow-up (Figure 3). Patients with 100% coverage of dispensed medicines experienced an average gain in CD4 count of 148.8 cells/mm3 (133.7, 164.0) between tests, compared to patients with <80% medicines coverage who gained an average 51.5 cells/mm3 (-25.6, 128.6). Patients with adherence from 80% to 100% experienced intermediate gains. Similarly, patients with a coverage gap longer than 30 days had gains in CD4 count that were an estimated 80.7 cells/mm3 less (-149.3, -12.1) than those without a gap.

**Table 5:** Estimates from general linear models of indicators of adherence and appointment keeping as predictors of change in CD4 count at time of follow-up CD4 test between 4 and 9 months after ART initiation.

	**Parameter estimate**	**P-value**	**Confidence interval**
			
Coverage (vs. 100% coverage) *			
<80% of days covered	**-97.3**	**0.0156**	**-176.1, -18.5**
80% to <90% of days covered	-28.8	0.2817	-81.2, 23.7
90% to <100% of days covered	-22.1	0.1224	-50.2, 6.0
Gap >30 days (vs. no gap in coverage)	**-80.7**	**0.0212**	**-149.3, -12.1**
Average self-report (vs. all self-reports perfect) ** &			
Better than good but less than perfect	-27.0	0.1752	-66.1, 12.1
Good or worse	**-67.2**	**0.0141**	**-120.8. -13.7**
Any self-report less than perfect (vs. all perfect) &	**-40.2**	**0.0186**	**-73.6, -6.8**
<80% of visits on day scheduled	-7.3	0.6759	-41.7, 27.1
<80% of visits within 3 days of schedule	-25.5	0.3605	-80.3, 29.3
<80% of visits before medicines finished	-8.6	0.5091	-34.1, 16.9

Key: All models use variables measured at the time of the follow-up CD4 test. For all models except those involving self-reported adherence, n = 401 cases; in all models involving self-report, n = 296 cases. Models include: gender, age category (30 or younger, 31-40, >40 years), whether married, capital-urban-rural location, baseline CD4 level, WHO stage at ART initiation (stage 1 or 2 vs. stage 3 or 4), time since ART initiation (in nine 30-day categories up to 270 days), evidence of TB at ART initiation, any evidence in medical record of side effect or opportunistic infection since treatment initiation, number of different ART regimens since initiation, ever treated with a protease inhibitor.

* F-test for inclusion of 3 coverage terms in model, p = 0.0465

** F-test for inclusion of 2 self-report terms in model, p = 0.0316

& Models exclude cases from Ethiopia which assessed self-reported adherence differently

Average self-reported adherence recorded in medical records up to the date of the second CD4 test had an overall significant relationship with CD4 gain. Compared to patients who always reported perfect adherence, gains in CD4 counts among patients who averaged between good and perfect self-reported adherence were a non-significant 27.0 cells/mm3 less (-66.1, 12.1), while CD4 gains among patients who averaged good self-reported adherence or worse were 67.2 cells/mm3 less (-120.8, -13.7), a difference which was significant. Patients with any report of less than perfect adherence experienced gains in CD4 count that were 40.2 cells/mm3 lower (-73.6, -6.8) than those who always reported perfect adherence.

The visit-based attendance measures were not significantly associated with improvements in CD4 counts.

## Discussion

This validation study shows that adherence and attendance indicators measured using routine data that exist in typical African HIV/AIDS care settings were significantly associated with key clinical outcomes during the early treatment period. Consistency of clinic visits and dispensing-based adherence indicators were both moderately to highly associated with weight gain during the first nine months after ART initiation. In addition, both dispensing-based adherence measures and self-reported adherence were predictive of improvements in CD4 counts in that period. Given that the data were extracted from routine records and are likely to contain random errors, the strengths of these relationships are likely to be underestimated.

In our study, the questions used to determine the adherence levels that were recorded in medical records were unknown and likely inconsistent across providers and treatment centers; in addition, self-reported adherence was recorded differently in Ethiopia than in the other three countries, requiring us to drop these cases from statistical analysis. Nevertheless, self-reported adherence measures showed promise. When measured in research settings, self-reported adherence has been reliably predictive of positive clinical outcomes[2-4,9]. Attention should be paid to strengthening the standardization of the recording of self-reports in routine records. Self-reported adherence measures (particularly indication of less than perfect adherence (i.e., missing any doses in a recent period, which is relatively easier to assess) would be useful both in clinical management of individual patients and in monitoring adherence in patient populations.

Our study has several notable limitations. First, we could not examine the validity of the INRUD-IAA indicators as predictors of patient dropout because study inclusion criteria required patients to remain in treatment for 4 to 6 months. This requirement was needed in order to obtain sufficient data to measure the dispensing-based and attendance-based measures, which depend on data from multiple visits. Most adherence failures occur early in the course of treatment. Our methods preclude examining the predictors and impacts of these early adherence failures.

We measured the validity of the adherence measures in predicting clinical changes soon after the start of therapy. Further analyses would be needed to assess the predictive validity of these measures for patients in treatment for longer periods. We also were unable to assess body mass index as a measure of nutritional status at the initiation of therapy, a potential confounder for post-initiation weight gain, due to the lack of data on height in the clinical record. Although food supplements offered at clinics would be another potential confounder of the relationship between adherence and early weight gain, we are not aware of any supplements offered at study facilities during the study period.

We did not assess the consistency of self-reported adherence in clinical and pharmacy records and as gathered by trained interviewers using a systematic method. These validation analyses will be part of future studies of the INRUD-IAA adherence indicators.

Despite these limitations, our study is the first to demonstrate that standardized measures of treatment adherence and attendance derived from routine data in African ART treatment settings are valid predictors of clinical outcomes among newly treated patients. Because these data are already available in most settings, they should be more widely used for monitoring adherence to ART and evaluating interventions.

## Conclusions

This study demonstrates that adherence measures derived from dispensing data in pharmacy records, self-report data in medical records, and attendance logs predict key clinical outcomes related to individual patient success in treatment. However, a more important use would be as population measures to characterize the overall success of treatment programs or health facilities in maintaining patients on therapy. With such data, it would be possible to target quality improvement activities to programs, facilities, and ultimately patients that are in greatest need.

## Competing interests

The authors declare that they have no competing interests.

## Authors' contributions

DRD and AW designed and planned the research, participated in design of the questionnaires, selected facilities for the study, analyzed the data, and drafted the manuscript. JC designed and planned the research, and participated in design of the questionnaires. MPJ cleaned the data, and assisted in analyzing the data. FZ assisted in analyzing the data. TH, LG, JN, and RB participated in design of the questionnaires, trained data collectors in their respective countries, and collected and entered the data. All authors read and approved the final manuscript.

## Pre-publication history

The pre-publication history for this paper can be accessed here:

http://www.biomedcentral.com/1472-6963/10/42/prepub
